# The Value of High Fusing Porcelain in Contour Work

**Published:** 1899-11

**Authors:** Joseph Head

**Affiliations:** Phila., Pa.


ARTICLE V.

### THE VALUE OF HIGH FUSING PORCELAIN IN CONTOUR WORK.

JOSEPH HEAD, M. D., D. D. S., PHILA., PA.

In the year 1882 Herbst was advocating glass fillings. These were made by taking impressions of the cavity in wax and making two moulds in such material as plaster and asbestos. The ground glass was then flowed into the first mould, where most of the shrinkage occurred. The partially formed filling was then removed, and placed in the second mould, when more glass was added until the filling was completed.

Even with this crude method the results were fairly satisfactory; but in 1887 Dr. C. H Land made mechanically perfert edges possible, by devising the metal matrix. He used both gold and platinum, but found the latter preferable, as platinum could be adapted with a facility equal to gold, and allowed the use of high fusing tooth body, which is much stronger and less likely to deteriorate than the lower fusing bodies, these of necessity containing a large percentage of borax or glass.

From the discovery of the metal matrix dates all effective porcelain filling. Pieces of porcelain had been ground to fit labial cavities with fairly good results, pieces of enamel from extracted teeth had been inserted in a similar fashion, but the accurate adaptation of porcelain to approximal cavities as far back as the molars was impossible until the metal matrix was evolved; and Dr. Land deserves the thanks of the profession for this discovery.

At present the value of porcelain fillings is being gradually recognized. But the advocates of this method are represented by two distinct factions, namely, those who believe in using a low fusing porcelain that will melt in a gold matrix, and those who believe in using a platinum matrix and a high-grade porcelain that has a fusing point at least equal to the continuous gum which has been provd through years of experience to be strong and permanent. Let us examine the advantages and disadvantages of these two methods. In either case the cavities are similarly prepared. They must be shaped free from undercuts so that the matrix can be readily withdrawn. Where necessary a large separation should be obtained, and this is usually necessary in approximal cavities. The edges should be sharp, they should be smooth and free from all irregularities. But here the similarity ends. The advocates of the gold matrix are either compelled to invest the matrix, to prevent the gold from being burnt, while the porcelain is being fused, or else to use a porcelain of such low fusing material as to make its future deterioration in the mouth almost a certainty.

On the other hand, the advocates of platinum can flow high fusing durable body into a bare matrix, which is clean and readily replaced in the cavity for a second burnish, without danger of the distortion that must of necessity arise if any of the investment adheres to tne outside.

If, however, but one burnish is used and the matrix is to be maintained in the investment until the entire filling is fused and ready for the cement, two objections may be noted that do not appear where the matrix is free to be replaced in the mouth. First, the color cannot be tested, and where necessary altered to a more perfect shade; second, the distortion caused by the unequal expansion and contraction of the metal and porcelain cannot be remedied as is possible when a second burnish is used.

Of course, to a great artist who could infallibly mix the colors to match, the first time, this chance for a second matching is unnecessary; but for the ordinary dentist such extra aids are valuable. In regard to the distortion caused by the unequal expansion and contraction of the matrix and porcelain, it is quite true that in pinhead cavities the warping is so slight that a second burnish is unnecessary, as the variation is less than the probable personal error of the operator; but in large cavities, especially where compound curves exist, the double burnish is most valuable, and the best results cannot be obtained without it.

Dr. Williams, in his interesting letter on porcelain fillings, that appeared in the April "Cosmos," advocates a high fusing body as a basis, and uses a lower fusing enamel for a finished surface. This is an excellent plan, and no doubt makes a much stronger filling than if the low fusing enamel only were used. I warmly approve his plan, and may add that I have used a method similar to it for over a year with good results. First, I used chips of high fusing porcelain teeth, mixed with low fusing enamel for the first biscuit. Later I used different colored high and low fusing bodies for the first and second bakings, just as Dr. Williams suggests, only my lowest fusing body was probably higher than his highest fusing body, but finally I decided to mix one part of high fusing colorless tooth body with three or four of the color desired in the lower fusing enamel for the first baking, and thus obtained excellent results without the necessity of having a large number of colors in the high fusing material. The one part of the high fusing colorless body added to three or four parts of the regular enamel lightened to so little that the plain enamel flowing over it on the second baking gives absolutely unchanged color, while undue contraction is avoided and the greatest strength obtained.

The plan advocated by Dr. Williams and others of packing in body while the matrix is in the tooth is excellent in simple cavities, easy of access, but in approximal cavities, complicated by blood and saliva, the matrix usually needs to be washed or heated to redness before any wet powder can advantageously be inserted. Theoretically the idea is excellent, and those who can successfully use it will no doubt be taking a step in advance. These slight deviations from Dr. Williams' method are, however, insignificant, and I heartily endorse his method and hope that the low fusing enamel used by him and Dr. Jenkins will prove as durable as the higher fusing enamels.

In summing up the pros and cons of the two schools, it may be noted that the advocates of gold for a matrix claim for it the sole, and if true, the very important, advantage that it makes a more perfect matrix than platinum.

While the advocates of platinum for a matrix feel that they are able to get perfect adaptation with it, they do not feel compelled to resort to investment; they are absolutely free in the use of the second burnish, and above all, they can fuse a material which resolutely keeps its color under fire, that makes contour work easy, and has undisputed strength and permanence.

A few words on the subject of furnaces may not be out of place. The gas furnaces of Land or Downie, with their platinum muffles, are most effective, in that the work can be removed without cooling the furnace. The highest fusing bodies can be baked in these furnaces, and if efficacy were all that is desired, these furnaces would be perfect, but they entail the working of the bellows by an experienced person; they necessitate a certain, or rather uncertain, amount of smoke and gas, and the noise that accompanies perfect combustion is disagreeable to the ears of nervous patients. Therefore, if the dentist can obtain a 110-volt current I should strongly advise him to do so; if, however, electricity be not obtainable, I should still advise him to get a gas furnace and to do his fusing in the laboratory. The little electric furnace manufactured by Ash & Son, so warmly advocated by Dr. Williams, is absolutely perfect for the purpose of which it was built; it fuses the low fusing bodies of Ash with great precision, and will not burn them unless there be great carelessness. It will not satisfactorily fuse the higher fusing bodies, such as are commonly used in the platinum matrix, but as it was not intended for that purpose this is no discredit, and it has the great advantage seldom possessed by electric furnaces of having an open muffle, like the gas furnace, from which the work can be removed without breaking the current or materially reducing the heat. The Custer electric furnace is the one I have found most satisfactory for high fusing work. The peep hole allows the work to be readily seen, and if a second or third baking is desirable, the combined time of fusing does not occupy over six or seven minutes.

Before closing, it may be well to clearly define the method of filling teeth with porcelain used by me.

The cavities, as before stated, must be free from undercuts, the edges sharp and smooth, and where they are approximal there must be sufficient separation to allow the matrix to be withdrawn with perfect freedom. This last is most important. Approximal cavities between the front teeth, as Dr. Williams urges, should be cut away freely from the back, just as we have long been accustomed to do in using gold. When the fillings are between bicuspids, the palatal or lingual walls should be cut away freely wherever it is necessary. With a perfect matrix excellent results can easily be obtained; with a distorted matrix success is impossible. Where the filling is to stand the force of mastication the edges of the cavity should always be at right angles to the grinding surface.

When a cavity is so prepared that the matrix may readily be removed, a piece of soft platinum one-thousandth of an inch in thickness that has been thoroughly annealed in an electric furnace or in a platinum muffle where no gas can touch it, is placed immovably over the cavity and burning into all parts, great care being taken to sharply and smoothly define the edges. Should the bottom tear, it is of little moment, so long as the edges are smooth and intact.

Sometimes it is useful to form the matrix by driving the metal into all recesses with cotton, but while this is undoubtedly of use in some instances, the careful burnishing of a motionless piece of foil will generally give the best results. The matrix when finished should be carefully removed, and in order to destroy all organic material heated to redness.

The high fusing porcelains made for inlay work by the Consolidated Dental Manufacturing Co. are in my opinion the best porcelain ever offered to the profession. They are strong, they are tough, and in addition blend their shades with the accuracy of water colors; and the color is so permanent that excessive heat modifies it but little. The color or colors having been chosen, by means of the shade ring, the corresponding enamels should be mixed with water on a glass slab to the consistency of dough. With contours and labial cavities an exact match should be obtained. with approximal cavities the filling should be considerably lighter as the shadows in between the teeth tend to make the dark color, and for want of this precaution disappointment awaits many an operator who has observed the greatest care and nicety in obtaining his match. In such approximal cavities pure white, toned with a little yellow, will in nineteen times out of twenty be the color needed.

When the enamel is mixed, according to the judgment of the operator, in small or simple cavities it can be used plain for both bakings, but in large cavities or contours, for the first baking, one part of colorless high fusing tooth body should be mixed with three parts of the enamel; this will make contour work easy, prevent undue contraction, and lightens the color so imperceptibly that the shade in the enamel in the second baking will be unchanged.

This extra high fusing mixture should be placed in the cavity almost, but not quite, up to the edges. If a contour is desired it should be formed then, proper allowance being made for the enamel that it is to be flowed over it later, but under all circumstances the edges must be left clean and free.

The matrix containing the body is then placed upside down on a piece of soft cotton cloth, to remove the excess of moisture which a few taps on the pliers holding the matrix will bring to the surface. It is then dried and placed in the furnace and fused to a medium gloss, then removed from the furnace, cooled, placed once more in the cavity, and the edges burnished to overcome any distortion caused by the unequal expansion and contraction of the metal and porcelain. The filling is then removed, and built up flush with the plain enamel, when the final firing is given. Should one or two firings be necessary to obtain perfect edges, they can be given, but only one extra burnish is necessary. In stripping off the matrix the edge should always be pulled first from the porcelain, otherwise chipping may result. Some dentists advise the use of the hydrofluoric acid for roughening the under surfaces of fillings before inserting them with cement; but fine grooves cut with a thin copper disk charged with diamond dust give better results.

Any thin, slow setting cement may be used for setting the porcelain. The color should approximate that of the filling. Before insertion the porcelain filling should be washed in alcohol and dried thoroughly, the cavity should then be slightly undercut and dried with alcohol; either a rubber dam or napkin can be used at this stage, but not sooner, as the tooth if dried before the filling is made will change its color, and so render the ultimate match bad. When everything is dry, the cement mixed to a creamy consistency should be placed in the cavity, and the filling being picked up on the end of a sticky spatula, pressed home. Pressure should be exerted until the cement sets or the expansion of the crystallization will cause gaping seams. A hot instrument applied to the inserted filling hastens the hardening of the cement very satisfactorily.

When the excess of cement is wiped off, paraffine or varnish should be flowed over the seams to protect the cement from the saliva, for at least six hours.

On the following day the filling may be dressed down with fine corundum stones or sandpaper disks.

This is the bare process. There are many minor though essential details that practice alone can acquire, details that are too numerous to dwell upon without the risk ot complexity. If, however, there are any points not clear, I hope the gentlemen present will mention them, and I shall cheerfully answer to the best of my ability. In closing I wish to emphasize this one fact. I have criticised the methods of some of my professional brothers in this field, but I do not wish it to be understood that I myself am beyond criticism. Too often in dentistry, as in other professions, personal feeling and petty jealousy interfere with scientific co-operation. I admire and respect the champions of low fusing porcelains for the work that they have done; but probably no method is at present perfect. The process of filling with porcelain is too young; time and experiment alone can choose the best, and we may fearlessly await time's decree, certain that whatever is truly best will in the end survive.—*Items of Interest.*

